# Welfare of Pigs Being Transported over Long Distances Using a Pot-Belly Trailer during Winter and Summer

**DOI:** 10.3390/ani4020200

**Published:** 2014-04-25

**Authors:** Jorge A. Correa, Harold Gonyou, Stephanie Torrey, Tina Widowski, Renée Bergeron, Trever Crowe, Jean-Paul Laforest, Luigi Faucitano

**Affiliations:** 1 F. Ménard Inc., Ange Gardien, QC, J0E 1E0, Canada; 2 Département de sciences animales, Université Laval, Quebec City, QC, G1K 7P4, Canada; E-Mail: Jean-Paul.Laforest@fsaa.ulaval.ca; 3 Prairie Swine Centre, Saskatoon, SK, S7H 5N9, Canada; E-Mail: hgonyou@shaw.ca; 4 Dairy and Swine and Development Research Centre, Agriculture and Agri-Food Canada, Sherbrooke, QC, J1M 0C8, Canada; 5 Department of Animal & Poultry Science, University of Guelph, Guelph, ON, N1G 2W1, Canada; E-Mail: twidowsk@uoguelph.ca; 6 University of Guelph, Alfred Campus, Alfred, ON, K0B 1A0, Canada; E-Mail: RBergeron@alfredc.uoguelph.ca; 7 Department of Mechanical Engineering, University of Saskatchewan, Saskatoon, SK, S7N 59, Canada; E-Mail: trever.crowe@usask.ca

**Keywords:** blood metabolites, heart rate, pigs, pork quality, season, transport

## Abstract

**Simple Summary:**

The pot-belly trailer, which can transport large loads in a single journey, is commonly used for swine transportation in Canada. However, it is generally acknowledged that pot-belly trailers and some specific compartments within this vehicle are worse than others in terms of animal losses. The objective of this study was to evaluate the effects of pot-belly design on animal welfare and meat quality in pigs being transported long distance in two different seasons of the year.

**Abstract:**

A total of 2,145 pigs were transported for 8 h in summer (six trips) and winter (five trips) using a pot-belly trailer accommodating pigs in four locations (upper deck or UD, bottom-nose or BN, middle deck or MD and bottom deck or BD). Heart rate of pigs during loading and transportation and lactate and creatine kinase (CK) concentrations in exsanguination blood were measured. Meat quality was evaluated in the *Longissimus thoracis* (LT), *Semimembranosus* (SM) and *Adductor* (AD) muscles. During summer, pigs loaded in the UD and MD had higher (*P* < 0.05) heart rate at loading compared to those located in the BD and BN. Blood lactate and CK concentrations were higher (*P* < 0.001) in winter than in summer. Lactate concentration was higher (*P* = 0.01) in the blood of pigs transported in the BN. Pigs transported in the BN had higher pHu values in the LT, SM and AD muscles (*P* = 0.02, *P* < 0.001 and *P* = 0.002, respectively) and lower (*P* = 0.002) drip loss values in the SM muscle. This study confirms that some locations within the PB trailer have a negative impact on the welfare of pigs at loading and during transport with more pronounced effects in the winter due to the additive effect of cold stress.

## 1. Introduction

In Western Canada, the large extension of the territory coupled with the centralization of the slaughter industry, with more pigs being killed in fewer larger plants, forces producers to transport pigs for long distances [1]. This has resulted in the increased use of pot-belly (PB) trailers that can transport large loads (up to 230 pigs) in a single journey. However, due to the presence of multiple steep internal ramps, pigs are more difficult to handle while loading and unloading this vehicle type [2], resulting in higher risk of animal losses during transport compared to other vehicle types [3,4]. In North America, a higher percentage of in-transit deaths and non-ambulatory pigs have been both recorded in summer (+0.15% and +2%, respectively) and in winter (+0.10% and +0.20%, respectively) compared to winter and summer, respectively [3,5,6,7]. These increases have been associated with the use of the PB trailer compared to a double-decked truck or a flat-deck trailer that are equipped with hydraulic decks [4,6,7]. Both cold and heat stress have an impact on *ante-* and *post-mortem* muscle glycogen stores leading to higher incidence of DFD (dark, firm, dry) and PSE (pale, soft, exudative) pork, respectively [8,9]. Recently, a greater percentage of PSE loins was found in the summer compared to winter (3% *vs.* 1%) and a greater incidence of DFD loins was reported in winter than in summer (8% *vs.* 4%) under Eastern Canadian transport conditions [4]. In two previous studies [4,10], the animal location (deck and/or compartment position in the truck) in the PB trailer during summer trasportation has an impact on pig body temperature and meat quality variation, with higher gastro-intestinal tract temperature in pigs in the upper deck and a greater percentage of pale pork being found in the upper and bottom decks compared to other locations. These effects are very likely the result of the physical exertion required by pigs to negotiate the ramps to get to these compartments under warm ambient conditions. However, these results were obtained in short distance transportation trials (2 h trip). Sutherland *et al.* [6] and Weschenfelder *et al.* [11] reported no detrimental effects on animal welfare when the PB trailer was used for a 9- and 7-h trip, respectively. However, in the former study [6] no interaction between trailer type and season was found, while in the latter one [11] these results were obtained under mild environmental conditions (11.2 °C on average). There is no evidence of the effects of the season and animal location on stress response and meat quality of pigs transported long distance in a PB trailer.

Because there is a gap in the knowledge on the role of ambient temperature and long-distance transportation on the variation of physiological variables and pork quality of pigs transported with a PB trailer, the objective of this study was to examine the physiological response (heart rate and exsanguination blood stress indicators) as well as skin bruise score and meat quality of pigs transported 8 h to slaughter in different transport compartments during both summer and winter.

## 2. Experimental Section

### 2.1. General

All experimental procedures performed in this study were approved by the AAFC Animal Care Committee in Sherbrooke (QC) based on the current guidelines of the Canadian Council on Animal Care [12]. A total of 2,145 crossbred pigs (BW = 115.2 ± 6.8 kg) were transported for 8 h (565 km) in summer (June and July, 2008; average temperature of 18.4°C, ranging from 9.1 to 20.7 °C) and winter (January to March, 2008; average temperature of –10.4°C, ranging from −22.3 to −9.7 °C) seasons from the Prairie Swine Center Elstow Research growing-finishing unit in Saskatchewan to a slaughter plant located in Brandon (Manitoba) using a dual-purpose (cattle and pig) pot-belly (PB) trailer.

**Figure 1:**
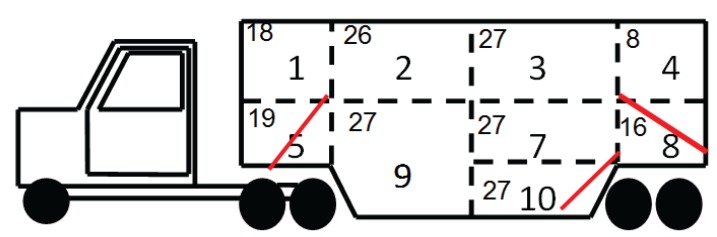
The location of compartments and distribution of pigs by compartment in the pot-belly trailer. Internal ramps are solid lines in compartments 5, 8, and 10.

Pigs were transported in 5 weeks in winter and 6 weeks in summer, in terms of one load of 195 pigs per week in each season, at an average density of 0.42 and 0.41 m^2^/pig, respectively. During the experiment, the transport schedules changed between the seasons to concur with the current transport practices of the region. In summer, the transporter only had two short stops (15 and 30 min each) after an initial transport phase of 190 min. These stops were considered short enough to protect pigs from the temperature and humidity rise inside a stationary PB trailer [11]. In winter, the transporter only had a single stop, lasting 180 min, before continuing the journey to the abattoir. Pigs were transported on three decks and distributed into 9 out of 10 compartments (four on the upper deck, three in the middle deck and two in the bottom deck of the vehicle; Figure 1). Compartment 6 was not filled due to load limitations. The trailer included three internal ramps: a 22° ramp going to the upper level (compartments 1, 2, 3 and 4), a 32° ramp going to compartment 5 (“bottom-nose” or BN), and a 22° ramp giving access to the bottom level (compartments 9 and 10; Figure 1). The trailer was bedded with wood shavings in the summer and straw and wood shavings in the winter. The side panels were open 100% in the summer, but only 10% in the winter. The trailer compartments were loaded in the following order: 5, 1, 2, 4, 3, 9, 10, 7, and 8. At loading, pigs were moved along an alley, up the chute into the trailer and then through ramps in groups of four or five using boards and the electric prod as necessary. The loading crew did not change between seasons.

Within a group of six pigs in each compartment, one pig (30 pigs/trip or replicate: total of 330 pigs) was chosen for physiological evaluation (heart rate and blood analysis). This pig plus another one selected out of every four pigs (45 pigs/replicate: total of 495 pigs) within the same group were also used for the meat quality assessment. Only barrows were chosen for these evaluations. Feed was withdrawn from the pigs 5–6 h prior to transport. Pigs were transported for 8 h and unloaded at the plant using boards and electric prods as necessary. After 1.5 to 2 h in lairage, pigs were driven in a single line to stunning, were electrically stunned (head-to-chest electrical stunning) and exsanguinated in the prone position.

### 2.2. Physiological Measurements

Heart rates were recorded by Polar heart rate monitors (Polar Electro Canada) at 5 s intervals for the duration of loading and transportation. For protection and stable positioning, heart rate monitors were covered by leather or nylon weight-lifting belts buckled around the pig’s chest. These were installed 24 h prior to transportation to allow animals to recover from the stress of this handling procedure. Belts were removed immediately after unloading. Data were downloaded and the average heart rate for each pig was determined for each of the experimental periods up to and including unloading.

At exsanguination, 2 mL of blood were collected in a tube (BD Vacutainers^®^, VWR International Ltd., Montreal, Canada) containing 6 mg of NA_2_ EDTA and 3 mg of NaF solution to extract plasma for lactate analysis and 10 mL of blood were put in a tube (BD Vacutainers^®^, VWR International Ltd., Montreal, Canada) to extract serum for creatine-kinase (CK) analysis. The 2-mL blood tubes were immediately centrifuged at 4 °C for 10 min at 1,400 × g. Plasma was then transferred into 1.5 mL Eppendorf tubes and stored at −80 °C until lactate determination. Serum samples were kept at room temperature (~23 °C) for 1 h before refrigeration at 4 °C. The following day, serum samples were centrifuged at 4 °C for 10 min at 1,400 × g, transferred to 1.5 mL Eppendorf tubes, and stored at −80 °C until analysis. Plasma lactate concentrations were measured with a microplate reader using a commercially available kit (Lactate Assay Kit, Biomedical Research Service Center, University of Buffalo, Buffalo, NY, USA) whereas CK concentrations were measured with a spectrophotometer using a creatine kinase-sl kit (Creatine Kinase-SL Assay of Chemicals Diagnostic Limited, Vancouver, Canada). All analyses were done in triplicate. The intra-assay coefficients of variation for CK and lactate concentration data were 3.43 and 3.40%, respectively.

### 2.3. Measurement of Carcass Quality Traits

Following slaughter, carcasses were eviscerated, split and blast chilled for 2 h. Hot carcass weight (HCW) and lean meat yield were recorded to characterize the population under study.

Skin damage was assessed on the day of slaughter in the cooler using the 5-point, photographic scale (1 = none to 5 = severe) [13], whereas bruises were classified as fighting-type bruises (score 1 = less than 5 bruises; 2 = 6 to 10 bruises; and 3 = greater than 10 bruises) and mounting-type bruises (score 1 = less than 5 bruises; 2 = 6 to 10 bruises) by visual assessment of shape and size according to the photographic standards of the Institut Technique du Porc [14]. According to the ITP scale, bruises due to biting during fighting are recognized as being of 5–10 cm in length, comma shaped and concentrated in high number in the anterior (head and shoulders) and posterior (ham) regions of the carcass. Long (10 to 15 cm), thin (0.5 to 1 cm wide) comma shaped bruises densely concentrated on the back of pigs typically caused by the fore claws were classified as mounting-type bruises. Lacerations and scratches normally produced when pigs are handled aggressively and run in closed and tight spaces were also noted and classified as “other bruise types”.

### 2.4. Meat Quality Measurements

Measurements of pH were made at 6 h *post-mortem* (after blast chilling) in the *longissimus thoracis* (LT) muscle at the third/fourth last rib level and *Semimembranosus* (SM) muscle and at 24 h (pHu) *post-mortem* in the LT, SM and *Adductor* (AD) muscles using a temperature-compensating, spear-type probe (Cole-Palmer Instrument Co., Vernon Hills, IL, USA) attached to a pH meter (pH 100 series; Oakton Instruments, Vernon Hills, IL, USA). At 24 h *post-mortem*, color data were collected on the LT and SM muscle after a 45 min bloom period. Visual color was evaluated using the Japanese color standards [15] in the LT muscle only, whereas instrumental color (L*, a*, and b* values) was measured with a Minolta Chromameter (CR-300; Minolta Canada Inc., Mississauga, Canada) equipped with a 25 mm aperture, 0° viewing angle, and D65 illuminant in the LT and SM muscles. Drip loss was measured in the LT muscle using the modified EZ-driploss method of Correa *et al.* [16]. Briefly, three 25 mm diameter cores were removed from the center of 2.5 cm thick LT (removed at the third/fourth last rib level) chop, weighed, and placed into plastic drip loss containers (Christensen Aps Industrivaengetand, Hilleroed, Denmark), before being stored for 48 h at 4 °C. At the end of the 48 h storage period, muscle cores where removed from their containers, surface moisture was carefully dabbed, cores were reweighed, and drip loss percentage was calculated by dividing the difference between initial and final core weights by the initial core weight.

### 2.5. Statistical Analysis

The experimental design was a one-way factorial (with the compartments as the levels of the factor to be compared) in a randomized complete block design (weeks as blocks) using the SAS software (SAS 2002) and repeated mixed model analyses (for heart rate data only) to test for any effects of season, compartment, transport event and their interactions. Considering the difference in transport schedule between seasons, for a reliable comparison only heart rate data recorded in the initial transport phase, which was of the same length in each season, were considered in the analysis. Carcass and meat quality data were analyzed by analysis of variance of SAS (SAS Institute Inc., Cary, NC, USA). A probability level of *P* < 0.05 was chosen as the limit for statistical significance in all tests.

## 3. Results and Discussion

No significant effect of the trailer compartment as a single factor or in interaction with the season was found on any physiological or meat quality variable (data not shown). Hence, data were pooled across compartments in order to study the effects of the deck (upper deck or UD = compartments 1+2+3+4; middle deck or MD = compartments 7+8; bottom deck or BD = compartments 9+10) or trailer section (bottom-nose or BN = compartment 5) on these variables.

**Figure 2:**
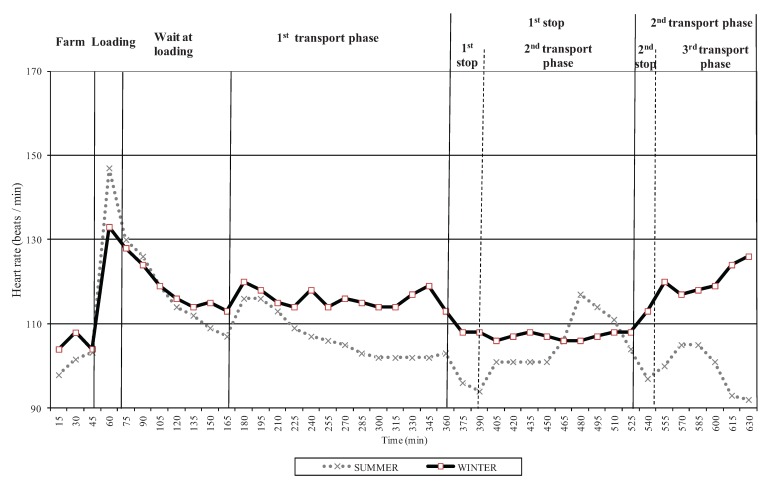
Heart rate from loading at the farm to 8 h transit time of pigs transported in a pot belly trailer in summer (six journeys) and winter (five journeys).

The significant differences are from the least square mean in every event, where at farm: *P* < 0.001; loading: *P* < 0.001; Transport: *P* < 0.001; first stop: *P* < 0.001. From the first stop, transport schedules changed between the seasons (statistical differences were not considered): in winter, first stop lasted 180 min and heart rate recording ends after the second transport phase, and in summer, first stop lasted 30 min. Second transport phase 140 min, second stop 15 min and heart rate recording ends after the third transport phase.

### 3.1. Physiological Response to Transport

As shown in Figure 2, heart rate was higher (*P* < 0.001) at loading in the summer, which may be explained by the increased frequency of slips, balks and vocalizations on the loading chute and truck ramp observed in this season compared to winter in a companion study [17]. A higher (*P* < 0.001) heart rate was also recorded during the first transport phase in winter. The increased heart rate in the initial transport phase during winter transports may be explained by the animal’s need to maintain the body temperature and to maintain balance while the truck was in motion as in a companion study pigs stood more at the beginning of the journey in winter than in summer transports [17]. More recently, Goumon *et al.* [18] reported higher heart rates and more pigs standing during the initial phase of transport using a similar PB trailer in winter. It is usually reported that pigs mostly stand in the initial phase of transport as they usually need time to acclimate in the truck after the departure from the farm [19,20].

An interaction was found between animal location in the PB trailer and season on pigs’ heart rate, with pigs in the UD and MD showing a greater heart rate during the wait after loading in summer (*P* = 0.01; Table 1). The higher heart rate recorded in pigs loaded on the UD may result from their physical effort to negotiate the steep ramp to have access to this location. Whereas, the loading order may explain the higher heart rates in pigs located in the MD as this was the last deck to be loaded and pigs were still under the effects of loading stress at the time of departure from the farm.

**Table 1:** Effects of animal location during transport on the pot-belly (PB) trailer on heart rate ^1^ average values in summer.

	Truck location^2^						
Transport phase	Total time (min)	UD	BN	MD	BD	SE	*P*-value
**N**		78	18	36	48		
**Farm**	120	95	96	98	96	2.19	0.74
**Loading**	5	152	154	147	146	5.23	0.54
**Waiting at loading**	120	124^a^	115^b^	129^a^	126^a^	2.61	0.01
**First transport phas**	190	109	106	109	104	2.32	0.29
**First stop**	30	95	97	94	95	2.09	0.88
**Second transport phase**	140	108	105	107	103	2.01	0.22
**Second stop**	15	97	94	97	96	2.57	0.93
**Third transport phase**	45	104	106	107	101	3.20	0.51

^1 ^Units of average values are in beats per minute; ^2 ^UD: upper deck (compartments 1,2,3,4); BN (compartment 5); MD (compartments 7,8); BD (compartments 9,10); ^a,b ^Within a row, least squares means lacking a common superscript differ at *P* < 0.05.

**Table 2:** Effects of animal location during transport in the PB trailer on heart rate average values ^1^ in winter.

	Truck location ^2^						
Transport phase	Total time (min)	UD	BN	MD	BD	SE	*P*-value
**N**		65	15	30	40		
**Farm (rest)**	120	107	102	103	105	2.06	0.27
**Loading**	5	135	139	128	133	2.96	0.15
**Waiting at loading**	120	120	126	125	122	2.86	0.25
**First transport phase**	190	118	118	112	116	2.20	0.15
**First stop**	180	108^b^	112^a^	102^c^	110^ab^	2.11	0.02
**Second transport phase**	90	122^a^	125^a^	114^b^	123^a^	2.84	0.06

^1 ^Units of average values are in beats per minute; ^2 ^UD: upper deck (compartments 1,2,3,4); BN (compartment 5); MD (compartments 7,8); BD (compartments 9,10); ^a,b,c ^Within a row, least squares means lacking a common superscript differ at *P* < 0.05.

Although a couple of companion papers reported a greater behavioral response (more slips and vocalizations) [17] to loading on the UD and colder temperatures in this deck during winter transports [21], heart rates of pigs did not differ between truck locations at any time in this season in this study (Table 2).

As no interaction was found for blood variables between season and animal location in the trailer, data were pooled and presented for each variable separately. Consistent with heart rates, both blood lactate and CK concentrations were higher (*P* < 0.001) in winter than in summer (Table 3). Higher lactate and CK concentrations are usually observed in blood of pigs subjected to rigorous physical exertion as a result of muscle tissue damage [22]. As such, both blood parameters are indicators of muscle fatigue, but in response to different types of stressors. Based on the relatively rapid rate of plasma lactate concentration increase (4 min) and return to basal levels (2 h) after physical exercise [23], the greater blood lactate concentrations at slaughter in winter than in summer may reflect the greater activity (longer latency to rest) in the lairage pen observed in a companion study in this season [17]. In this season pigs were thus more fatigued at slaughter as they stood more than lying in lairage and could not recover from transport and handling stress. Áveros *et al.* [24] and Seddon *et al.* [25] also found elevated exsanguination blood lactate concentrations in winter compared to summer.

**Table 3:** Lactate and creatine kinase (CK) concentrations in exsanguination blood of pigs (n = 329) transported on the PB trailer according to the season and the truck location.

	Season				Truck location ^1^						*P* –value	
	Summer	Winter	SE		UD	BN	MD	BD	SE		Season	Location
CK (UI/L) ^2^	4,605.8	6,865.8			5,695	5,892	4,760	6,327			<0.001	0.20
Lactate (mmol/L)	10.4	13.3	0.5		12.2 ^b^	14.2 ^a^	10.1 ^c^	11.9 ^b^	0.7		<0.001	0.01

^1 ^UD: upper deck (compartments 1,2,3,4); BN (compartment 5); MD (compartments 7,8); BD (compartments 9,10); ^2 ^CK: Creatine kinase (SE is not presented because CK data have been calculated with inverse transformation); ^a,b,c ^Within a row, least squares means lacking a common superscript differ at *P* < 0.05.

Higher (*P* = 0.01) blood lactate concentrations were also observed in pigs transported in the BN compartment. This increase may be associated to the response to *peri-mortem* handling in pigs having experienced a previous physical stress while negotiating the two ramps, of which one was very steep (32°), to access to this truck location [17].

Similar to blood lactate concentrations, blood CK levels were greater (*P* < 0.001) in winter than in summer. Correa *et al.* [4] also reported higher blood CK levels in winter than in summer. Differently from blood lactate, CK concentration in blood is strongly correlated with long-term physical stress as it reaches a maximum concentration peak 6 h after the physical effort and does not return to basal levels until 48 h afterward [23]. Based on its dissipation pattern in blood after stress, the higher blood CK concentrations in winter in this study may reflect the greater fatigue of pigs at slaughter in this season. In this study, fatigue may be associated to the higher incidence of slips and falls at loading recorded in winter [17]. All these events may have resulted in muscle tissue damage and increased CK release into the blood flow. Similar to Correa *et al.* [4], animal location within a PB trailer had no effect on blood CK concentrations at slaughter.

### 3.2. Carcass Quality Traits

On average, HCW of the population under study was 95.7 kg (ranging from 65.5 to 129.8 kg) and the carcass lean percentage was 61.2% (ranging from 55.1 to 66.6%).

Overall, skin damage score and the proportion of carcasses showing fighting-type bruises were higher (*P* = 0.02 and *P* < 0.001, respectively) in the summer transports compared to the winter ones (Table 4). The higher skin damage score in summer may be associated with the increased frequency of slips on the loading chute and truck ramp and slips, falls and overlaps observed at unloading in a companion study [17]. The reduced proportion of bruises in winter in this study contradicts the results from previous studies which either reported a higher incidence of bruised carcasses in this season due to increased fighting or mounting behavior in pigs huddling to maintain their body temperature [26,27] or which failed to find a seasonal variation in skin bruise scores [28].

**Table 4:** Skin damage score and percentage of bruised carcasses of pigs transported on the PB trailer according to the season and the truck location.

	Season				Truck location ^1^						*P*-value	
	Summer	Winter	SE		UD	BN	MD	BD	SE		Season	Location
**N**	257	215			185	55	104	129				
**Skin damage**^ a^	2.3	2.1	0.046		2.2	2.3	2.1	2.2	0.1		0.02	0.42
**Fighting type bruise**^ b^**, %**	46.5	24.7			36.8	38.2	35.6	35.7			<0.001	0.80
**Mounting type bruise**^ c^**, %**	0.8	1.4			1.1	0.00	1.0	1.5			0.99	0.68
**Other type of bruises**^ d^**, %**	21.3	21.9			23.2	21.8	17.3	23.3			0.96	0.75

^1 ^UD: upper deck (compartments 1,2,3,4); BN (compartment 5); MD (compartments 7,8); BD (compartments 9,10); ^a ^Based on the MLC scale from 1 (none) to 5 (severe) [13]; ^b ^Percentage of carcasses with more than 10 fighting-type bruises according to the ITP scale [14]; ^c ^Percentage of carcasses with more than 5 mounting-type bruises according to the ITP scale [14]; ^d^ Percentage of carcasses presenting bruises of different origin (lacerations, scratches, *etc.*).

The greater proportion of fighting-type bruises recorded in the summer compared to winter is hard to explain considering that pigs laid more during transport and took shorter time to rest in the lairage pen after transport in summer compared to winter [17].

Despite the variation in pig postures observed between truck compartments at unloading, with pigs from the top compartments being slower to move out and those from the bottom compartments overlapping more [17], bruise score did not vary between truck locations in this study. This discrepancy between pigs’ posture observation in the truck and bruise scores has been also reported by Scheeren *et al.* [29] using a similar PB trailer model.

### 3.3. Meat Quality

It is known that cold and heat stress have an impact on *ante*- and *post-mortem* muscle glycogen stores leading to higher incidence of DFD and PSE pork, respectively [8,9]. As expected, pH24 values were greater (*P* < 0.001) in the LT, and SM and AD muscles in winter than in summer (Table 5). Consistently, in winter the LT muscle had lower (*P* = 0.001) drip loss and higher (*P* = 0.02) subjective color score (darker color). However, in the SM muscle pH6 value was lower (*P* < 0.001) and drip loss value was higher (*P* = 0.03) in winter than in summer. Both meat quality traits are indicative of increased muscle acidification which may result in PSE pork. O’Neill *et al.* [30] also reported a higher prevalence of PSE meat in winter compared to summer and hypothesized that it was related to faster slaughter rates in winter. To maintain fast slaughter speeds, handlers push pigs to walk at a faster pace resulting in acute physical stress immediately before slaughter. It is not surprising that this effect is more pronounced in a locomotory muscle, such as the SM, as this muscle is more susceptible to physical exercise [11,18,31].

**Table 5:** Meat quality variation as measured in the *Longissimus thoracis* (LT), *Semimembranosus* (SM) and *Adductor* (AD) muscles of pigs transported on a PB trailer according to the season and the truck location.

	Season				Truck location ^1^						*P*-value	
	Summer	Winter	SE		UD	BN	MD	BD	SE		Season	Location
N ***LT muscle***	270	225			198	55	110	132				
pH 6 h	6.02	6.02	0.02		6.04	5.99	6.02	6.00	0.03		0.84	0.75
pH 24 h	5.64	5.73	0.01		5.69 ^a^	5.70 ^a^	5.66 ^b^	5.66 ^b^	0.02		<0.001	0.02
L*	49.19	49.04	0.26		49.32	48.29	49.09	49.12	0.36		0.67	0.34
a*	8.43	8.16	0.09		8.34	8.16	8.30	8.25	0.13		0.03	0.81
b*	4.91	4.90	0.08		4.96	4.62	4.95	4.92	0.12		0.94	0.29
JCS ^2^	2.94	3.11	0.06		3.02	3.18	3.00	2.98	0.08		0.02	0.42
EC ^3^	6.99	7.49	0.17		7.57 ^a^	7.21 ^a^	7.20 ^a^	6.87 ^b^	0.23		0.03	0.04
Drip loss (%)	4.05	3.39	0.14		3.70	3.21	3.89	3.83	0.20		0.001	0.19
***SM muscle***												
pH 6 h	6.32	6.07	0.02		6.18	6.21	6.19	6.21	0.03		<0.001	0.86
pH 24 h	5.63	5.71	0.01		5.66	5.76	5.65	5.69	0.02		<0.001	<0.001
L*	46.84	46.19	0.21		46.86 ^a^	46.24 ^ab^	46.69 ^a^	45.97 ^b^	0.29		0.03	0.03
a*	7.90	8.64	0.10		8.28	7.85	8.47	8.27	0.14		<0.001	0.08
b*	3.69	4.08	0.07		3.96 ^a^	3.68 ^b^	4.01 ^a^	3.79 ^a^	0.09		<0.001	0.007
EC ^3^	6.88	6.98	0.18		7.17	6.62	6.86	6.67	0.26		0.68	0.30
Drip loss (%)	3.77	4.15	0.13		4.17 ^a^	3.76 ^b^	3.98 ^ab^	3.96 ^ab^	0.18		0.03	0.002
***AD muscle***												
pH 24 h	5.81	6.01	0.02		5.91^a^	5.97 ^a^	5.84 ^b^	5.92 ^a^	0.03		<0.001	0.002

^1 ^UD: upper deck (compartments 1,2,3,4); BN (compartment 5); MD (compartments 7,8); BD (compartments 9,10); ^2 ^According to Japanese Color Scales (from 1 = pale to 6 = dark) [15]; ^3 ^Electrical conductivity measured by the PQM (Pork Quality Meter, Aichach, Germany); ^a,b,c ^Within a row, least squares means lacking a common superscript differ at *P* < 0.05.

Meat quality was also affected by animal location during transport, with pigs located in the BN and UD having higher (*P* = 0.02) pH24 values (Table 5). Similar to the effects of the season, the effects of transporting pigs in the BN were more pronounced in locomotor muscles, with SM and AD muscles of these pigs showing higher (*P* < 0.001 and *P* = 0.002, respectively) pH24 values. Lower L* values and drip loss percentages (*P* = 0.03 and *P* = 0.002, respectively) were also reported in the SM muscle of pigs transported in the BN. These meat characteristics indicate that pigs loaded in the BN area may become fatigued and be prone to produce DFD pork.

## 4. Conclusions

This study shows that some pot-belly trailer locations, such as the upper deck and bottom- nose, impose a certain level of stress on pigs during transport resulting from the use of internal ramps at loading and unloading. Additional studies are needed to determine means of reducing the stress of moving pigs into and out of such trailer locations. Although in this study the effect of the season may have been confounded by the different transport schedule between seasons, which reflects the commercial practice in the Canadian Prairies, the greater physical exhaustion (higher blood lactate and CK levels, and pH24 values) of pigs transported long distance in winter which is due to the additive effect of cold stress confirms results that have been previously reported in the literature [4,25,32].
